# Electroporation and Microinjection Successfully Deliver Single-Stranded and Duplex DNA into Live Cells as Detected by FRET Measurements

**DOI:** 10.1371/journal.pone.0095097

**Published:** 2014-04-22

**Authors:** Rosemary A. Bamford, Zheng-yun Zhao, Neil A. Hotchin, Iain B. Styles, Gerard B. Nash, James H. R. Tucker, Roy Bicknell

**Affiliations:** 1 PSIBS Doctoral Training Centre, University of Birmingham, Birmingham, United Kingdom; 2 School of Chemistry, University of Birmingham, Birmingham, United Kingdom; 3 School of Biosciences, University of Birmingham, Birmingham, United Kingdom; 4 School of Computer Science, University of Birmingham, Birmingham, United Kingdom; 5 Centre for Cardiovascular Sciences, School of Clinical and Experimental Medicine, University of Birmingham, Birmingham, United Kingdom; 6 Institute of Biomedical Research, School of Immunity and Infection, University of Birmingham, Birmingham, United Kingdom; CNR, Italy

## Abstract

Förster resonance energy transfer (FRET) technology relies on the close proximity of two compatible fluorophores for energy transfer. Tagged (Cy3 and Cy5) complementary DNA strands forming a stable duplex and a doubly-tagged single strand were shown to demonstrate FRET outside of a cellular environment. FRET was also observed after transfecting these DNA strands into fixed and live cells using methods such as microinjection and electroporation, but not when using lipid based transfection reagents, unless in the presence of the endosomal acidification inhibitor bafilomycin. Avoiding the endocytosis pathway is essential for efficient delivery of intact DNA probes into cells.

## Introduction

DNA and its derivatives have the potential to detect, monitor and control the expression levels of specific genes in living cells in real time [1], which has led to interest in various therapeutic developments based on nucleic acids such as antisense treatments [2], [3] and gene screening [4]. The benefits of using DNA include its high selectivity and non-toxicity as well the relative ease with which various functional tags can be introduced for monitoring in a cellular environment. However there are several issues that need to be considered when using this approach, centred around firstly the most effective method for delivery and secondly the fate of the DNA once it is introduced into the cell. As far as the latter is concerned, several strategies [5]–[7] have been employed to mitigate factors such as susceptibility to degradation by nucleases [8], non-specific binding to proteins and unwanted migration to the nucleus if the interaction with non-nuclear targets is required [9].

The most popular technique for effecting non-viral delivery of DNA into cells (i.e. transfection as opposed to transduction) is to use chemical reagents that facilitate the passage of polyanionic DNA through the membrane bilayer [10]. DNA modification has also been shown to enhance cell delivery, with attached peptides facilitating chemical transfection [11], [12] and Locked Nucleic Acids (LNA) shown to have been taken up without the use of transfection reagents [13]. Another uptake methodology is microinjection, which has been used in a study comparing the cell stability of phosphodiester and phosphorothioate oligonucleotides [14], [15]. A common alternative to microinjection is electroporation, which uses a rapid and high-voltage electric pulse that causes pore formation in the membrane [16]. Nevertheless there is a sparsity of literature that compares different transfection methodologies and their possible effect on intracellular DNA stability. We decided to address this by embarking on a controlled fundamental study to compare the various techniques for transfection of DNA into cells, including invasive methods such as microinjection and electroporation, and non-invasive methods such as chemical transfection.

Fluorescence microscopy was chosen as the method for monitoring cell transfection through the use of fluorophore-tagged DNA strands [17], which is by far the most common way of tracking cellular processes *in vitro*. Doubly tagged single strands or duplexes were chosen to allow transfection to be monitored by Förster resonance energy transfer (FRET). FRET is the physical process that occurs when the excited-state energy of a donor fluorophore is transferred nonradiatively to an acceptor in the ground state [18], which results in quenching of the donor fluorophore and excitation of the acceptor. The efficiency of energy transfer depends on the spectral overlap of the emission and absorption spectra of the donor and acceptor respectively, as well as their respective distance and orientation. The distance dependence of FRET can monitor differences over the range of 10–100 Å, which is ideal for macromolecules such as nucleic acids [19], [20]. FRET can be used to detect and quantify sequences extracted from biological samples [21]–[23] including real-time PCR assays [24]–[26]. It has also been widely used to detect hybridisation of donor- and acceptor-labelled complementary nucleic acid strands [27]–[30]. This in turn can allow the integrity of a duplex to be monitored upon entry into the cell, which is relevant to this study. As for the choice of FRET pair, fluorophores Cy3 (donor) and Cy5 (acceptor) are commonly used in nucleic acid experiments due to their easy attachment to DNA, high FRET efficiency, relatively low photobleaching and long emission wavelengths away from the autofluorescence region of cells [31].

As described below, having confirmed that Cy3-Cy5-tagged DNA displays FRET in a cuvette in its single stranded and duplex form, a comparison of the effectiveness of delivery of intact DNA to cells using FRET is then described, via various techniques that include chemical transfection, microinjection and electroporation. The work demonstrates how the choice of technique is crucial for optimising the stability of DNA strands and duplexes in a cellular environment.

## Materials and Methods

Unmodified and tagged oligonucleotides were synthesised as previously described using the phosphoramidite method [32] (Applied Biosystems 394). Cy3 and Cy5 phosphoramidites (Glen Research) were tagged to the 5′ and 3′ termini. Deprotection was carried out using ammonia and ethanol at room temperature. Oligonucleotides were purified by reversed-phase HPLC and characterised by electrospray mass spectrometry. UV-vis spectra were recorded using a Shimadzu UV-Vis 1800 spectrophotometer.

Fluorescence spectra were recorded on a Shimadzu RF-5301 PC spectrofluorophotometer. The excitation wavelength was selected at 554 nm. The sample solutions were as follows: 10 mM sodium phosphate pH 7.0, 100 mM NaCl, and 1.0 µM each DNA strand. The melting temperature (*T*_m_) of duplex DNA was obtained on a Varian Cary-5000 by measurement of the change in absorbance at 260 nm as a function of temperature. The temperature ramp was 0.5°C min^−1^. The sample solutions for UV/Vis spectroscopy were as follows: 10 mM sodium phosphate, 100 mM NaCl, pH 7.0, 5 µM each DNA strand.

Chinese hamster ovary (CHO) cells were grown at 37°C in a humidified atmosphere of 5% CO_2_. Cells were maintained by regular passage in DMEM (Sigma Aldrich). The medium was supplemented with 10% heat-inactivated fetal bovine serum (FBS), 2 mM L-glutamine, 100 U/ml penicillin and 50 U/ml streptomycin (gibco by Life Technologies). To test the stability and specificity of the DNA, it was incubated at 37°C in cell lysate extracted from CHO cells, and fluorescence spectra collected at intervals over a 2 hour period. To test degradation, DNA was incubated with DNase I for 2 hours before being added to CHO cell lysate and the fluorescence spectra recorded.

For cell fixation, 3×10^5^ CHO cells were seeded in DMEM on Mattek dishes. The cells were fixed and permeabilised using −20°C methanol for 5–10 minutes. The cells were exposed to 0.05 mg/ml DNA in PBS for 1 hour and then rinsed with PBS solution. If DNA was added sequentially, the cells were exposed to the second strand for a subsequent 1 hour and then rinsed with PBS solution.

For chemical transfection, CHO cells were grown on Ø13 mm coverslips for 24 hours in complete DMEM. Transfection was carried out using 100 µM DNA, Opti-MEM medium (Life Technologies) and Lipofectamine RNAiMAX (Life Technologies). Transfection was carried out over 4 hours at 37°C. Cells were fixed with 4% formaldehyde and nuclei stained with Bisbenzamide (Sigma) for imaging purposes. Bafilomycin A1 (Sigma Aldrich) was dissolved in DMSO and added to the transfection medium (final concentration 100 nM) as above. Confocal images were acquired with a laser scanning 510-UV confocal microscope (Zeiss); Bisbenzamide (364 nm/351 nm laser, em BP 385–470 nm); Cy3 (543 nm laser, em BP 560–615 nm) and Cy5 (633 nm laser, em LP 650 nm). Beam splitter: MBS (HFT UV/488/543/633).

For microinjection, 1.5×10^5^ CHO cells were seeded in DMEM on Mattek dishes. Prior to microinjection, the medium was replaced with HEPES supplemented DMEM. Microinjection was performed using a micromanipulator (model 5171, Eppendorf) and transjector (model 5246 Plus/Basic; Eppendorf). A DNA concentration of 100 µg/µl was microinjected into the cytoplasm of cells.

For electroporation, 8×10^5^ CHO cells were added to serum free DMEM and 25 µg/ml DNA in a 4 mm gap electroporation cuvette (Geneflow) for 10 minutes at room temperature. Electroporation was carried out at 400 V and 25 µF (BioRad Gene Pulsar II). The cells were left for 5 minutes at room temperature and then for 5 minutes on ice. The cells were then seeded in DMEM on Mattek dishes and allowed to recover for 12 hours.

All cell imaging, excluding transfected cells, was carried out on an inverted confocal microscope (Zeiss); Cy3 (543 nm laser, MBS 488/543/633, em 515–613 nm) and Cy5 (633 nm laser, MBS 488/543/633, em 698–754 nm). Transfected cells were imaged on an axiovert UV confocal microscope (Zeiss); BB (364 nm, 351 nm laser, MBS UV/488/543/633, em BP 385–470 nm), Cy3 (543 nm laser, MBS UV/488/543/633, em BP 560–615 nm) and Cy5 (633 nm laser MBS UV/488/543/633, em LP 650 nm). Emission microscopy was carried out on a spectral imaging inverted confocal microscope (Leica); Cy3 (543 nm laser, MBS UV/488/543/633, em 556–615 nm) and Cy5 (633 nm laser MBS UV/488/543/633, em 641–750 nm).

For statistical analysis, data is plotted with error bars representing standard error of the mean. Emission intensity values were taken from ROI in cell images, with at least ten cells analysed. In order to compare Cy5 intensity values between using both the 543 nm and 633 nm lasers, and the 543 nm laser only, the Mann-Whitney test was performed. All calculations were performed offline using Matlab 2009a.

## Results

### Synthesis and Characterisation of DNA Probes

Table 1 shows the main oligonucleotides synthesised for this study (for non-complementary oligonucleotides see Table S1 in File S1). Tagged DNA strands were prepared by automated solid phase synthesis using conventional phosphoramidite chemistry, as reported previously [32]. Complementary strands **S1** and **S2** containing respectively a Cy3 and a Cy5 fluorophore at the 5′ terminus were prepared for duplex studies, in addition to a strand containing the fluorophores at each end (**S3**). Each strand was purified by reversed phase HPLC (Table S2, Figures S1-S5 in File S1) and characterised by mass spectrometry (Table S3 in File S1), with UV-vis melting studies confirming that the **S1**:**S2** duplex was stable at both room temperature and at 37°C in salt conditions appropriate for cell studies (10 mM NaCl) (Table S4 in File S1).

**Table 1:** Tagged oligonucleotides synthesized.

Oligonucleotide Name	Sequence (5′ to 3′)
Cy3 strand (**S1**)	Cy3-TGGACTCTCTCAATG
Cy5 strand (**S2**)	Cy5-CATTGAGAGAGTCCA
Cy3 and Cy5 strand (**S3**)	Cy5-TGGACTCTCTCAATG-Cy3

### Cuvette Fluorescence Spectroscopy

**Figure 1:**
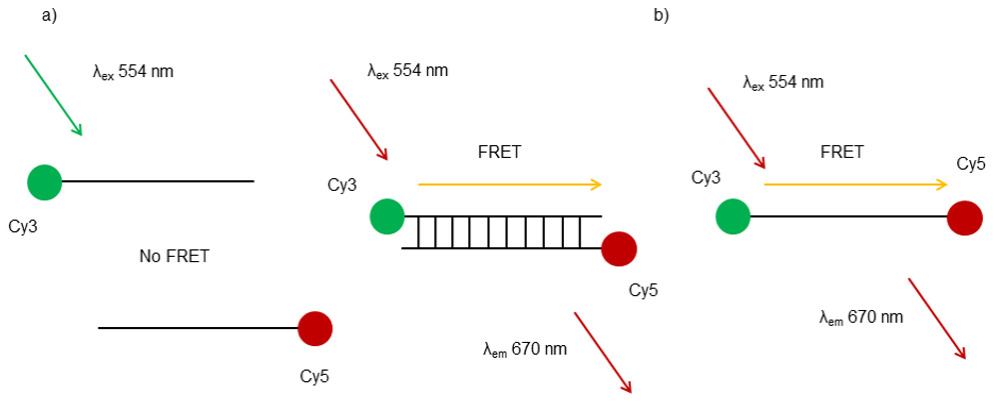
Schematic of Cy3 and Cy5 tagged DNA. a) Complementary DNA strands are individually tagged with Cy3 and Cy5 fluorophores (**S1** and **S2** respectively). When in close enough proximity the Cy3 can donate energy to Cy5 through FRET. In this case, FRET can only occur when the two complementary strands form a duplex. b) Single strand DNA can be tagged at either end with Cy3 and Cy5 (**S3**). FRET can occur as long as the single strand remains intact.

**Figure 2:**
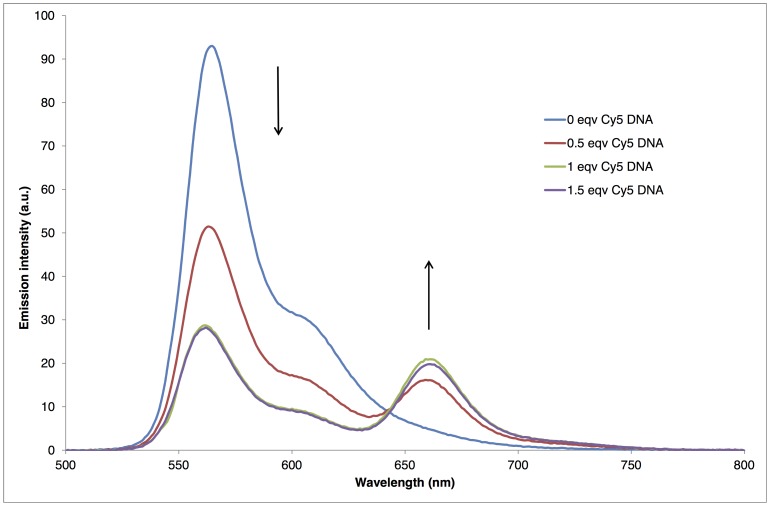
Emission spectra of Cy3 and Cy5 DNA. Titration of Cy5 tagged DNA (**S2**) into Cy3 tagged DNA (**S1**), showing resulting Cy5-Cy3 FRET upon duplex formation (excitation wavelength = 554 nm). The emission intensities centred at 570 nm and 670 nm correspond to emission from Cy3 and Cy5 respectively (conditions: 1 µM DNA, 100 mM NaCl, and pH 7.0 sodium phosphate buffer). The spectra are subtracted for the spectrum of **S2** alone, excited at 554 nm, which gave a small signal caused by direct excitation of the Cy5 chromophore.

For both strand **S3** and the **S1**:**S2** duplex, the Cy3-Cy5 fluorophore pair was expected to be in close enough proximity to display FRET (Figure 1). FRET was indeed evidenced by fluorescence spectroscopy studies in a cuvette (10 mM sodium phosphate buffer, 100 mM NaCl, pH 7.0, 1 µM each DNA strand) in which the emission intensity from the Cy3 and Cy5 tags was monitored over the range 500–800 nm, when exciting only the Cy3 chromophore directly. In particular a titration study involving the addition of **S2** to **S1** indicated that the Cy3 signal at 570 nm decreased, while the signal for Cy5 at 670 nm increased, with no further increases observed after the addition of one molar equivalent of the target, consistent with 1∶1 duplex formation (Figure 2). Control studies indicated little or no emission at 670 nm when **S2** was irradiated alone in the absence of **S1** at 554 nm under the same conditions. Similar results and trends were obtained for the doubly-tagged strand **S3**.

The FRET signal from the **S1:S2** duplex and **S3** were then studied in CHO cell lysate at 37°C in the absence and presence of DNase (Figures S6-S7 in File S1). In cell lysate alone, over a period of 2 hours, only small changes in the emission spectra were observed. However as expected, the addition of nuclease brought about a rapid decrease in the FRET signal for both systems, indicating backbone cleavage of the DNA in either its single-stranded or duplex form [30].

### Fixed Cell Fluorescence Microscopy

**Figure 3:**
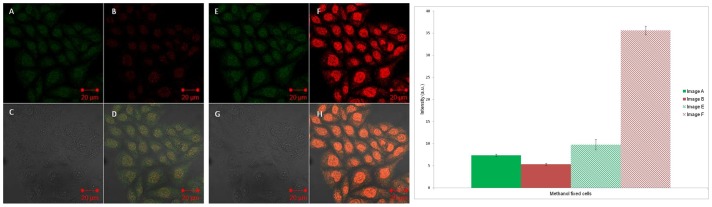
Fixed cell confocal microscopy images. Left: Cy3 and Cy5 tagged DNA duplex (**S1:S2**) added to fixed/permeabilised cells and imaged using confocal microscopy. Images A/E represents the Cy3 channel; B/F the Cy5 channel; C/G the bright field channel and D/H an overlay of all the channels. Images A–D are excited with the 543 nm laser. Images E–H are excited with both the 543 and 633 nm lasers. Right: Intracellular fluorescence intensity from images A/B and E/F. Data are expressed as mean ± s.e.m from at least ten cells (p<0.001).

Having observed the desired FRET effect under cuvette conditions, the same strands were then exposed to CHO cells that had previously been fixed using methanol, to allow the strands to readily permeate into the cell, which was otherwise not possible with live cells. The successful transfection of **S1**:**S2** as an intact duplex was evidenced by FRET (Figure 3) at room temperature using scanning laser confocal microscopy. The key result was the observation of a signal in Image B (Cy5 channel) upon excitation at the Cy3 absorption wavelength, with a control study indicating no emission observed under these conditions when fixed cells were transfected with **S2** alone (Figure S8 in File S1). Quantitative data extracted from the intensities of the cell images in Figure 3 also showed significant FRET based on the ratio between the Cy5 intensity and Cy3 intensity upon excitation at the Cy3 absorption wavelength only (Figure 3, first two bars on chart). The *in situ* formation of a duplex was also indicated by FRET when the strands were added sequentially (**S1** followed by **S2**) in order to replicate the cuvette experiment and show that the sequences were able to find each other in a cell environment (Figure S9 in File S1). A similar FRET signal was also seen on the addition of **S3** to fixed cells but as expected, non-complementary Cy3 and Cy5-tagged DNA strands, added either together or sequentially, were shown not to display FRET (Figures S10-S11 in File S1). To enable a closer comparison with the cuvette studies, emission spectra were also recorded in fixed cell samples using spectral imaging inverted confocal microscopy (Figure S12 in File S1), and these gave broadly similar profiles, confirming the presence of FRET in fixed cells within both the **S1:S2** duplex and the **S3** strand.

### Live Cell Fluorescence Microscopy

Whereas fixed cells could be readily transfected by simple exposure to a PBS solution of the modified DNA strands in their single stranded or duplex forms, as expected, established transfection methodologies were required to transfect live cells, as described below.

#### 1. Chemical Transfection

**Figure 4:**
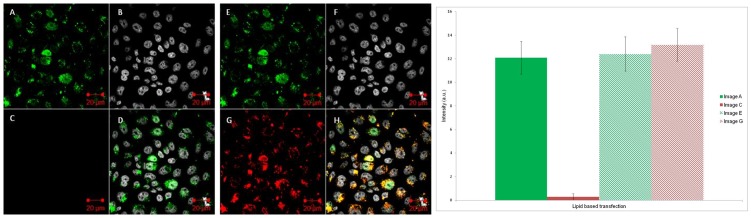
Chemical transfection confocal microscopy images. Left: Cy3 and Cy5 tagged DNA duplex (**S1:S2**) added to cells via chemical transfection using Lipofectamine and imaged using confocal microscopy. Images A/E represents the Cy3 channel; B/F the nuclear stain channel; C/G the Cy5 channel and D/H an overlay of all the channels. Images A–D are excited with a 543 nm laser only. Images E–H are excited with both the 543 and 633 nm lasers. Right: Intracellular fluorescence intensity from images A/C and E/G. Data are expressed as mean ± s.e.m from at least ten cells (p<0.001).

The preformed **S1:S2** duplex in PBS was treated with the chemical transfection agent Lipofectamine. FRET was still observed for the complex between DNA and Lipofectamine (Figure S13 in File S1) prior to incubation with CHO cells and visualisation by confocal microscopy as before. Once again, excitation of the Cy3 and Cy5 fluorophores at their respective excitation wavelengths indicated that they were both present within cells and co-localised. However this time when only the Cy3 laser was turned on, no Cy5 signal was observed, and hence no FRET was occurring (Image C, Figure 4). Quantitative data in Figure 4 clearly shows negligible Cy5 signal compared to Cy3 signal upon excitation at the Cy3 absorption wavelength only (Figure 4, first two bars on chart). Similar results were observed for the chemical transfection of **S3** (Figure S14 in File S1), which meant that the absence of FRET being ascribed to dissociation of the duplex in the cellular environment could be essentially ruled out. Emission spectra were also measured for chemically transfected cell samples using spectral imaging inverted confocal microscopy (Figure S15 in File S1), which confirmed the absence of a FRET signal under these conditions.

It was observed that the Cy3 and Cy5 fluorescence was to some extent co-localised in a punctate pattern rather than being evenly distributed. These results were consistent with the tagged DNA being unable to be released from endosomes once within the cell and subsequently digested by nucleases [12], [33], [34]. It is hypothesised that the tagged oligonucleotides, whether in their single strand or duplex forms, are being degraded within vesicles on entry to the cell *via* endocytosis.

When strands **S1** and **S2** were transfected into cells individually under these conditions, there was shown to be no crosstalk between the Cy3 and Cy5 channels, since upon excitation, only signals from their respective channels were observed (Figure S16 in File S1). As expected, non-complementary Cy3 and Cy5 oligonucleotides added together *via* chemical transfection were also shown not to display FRET (Figure S17 in File S1).

#### 2. Microinjection

**Figure 5:**
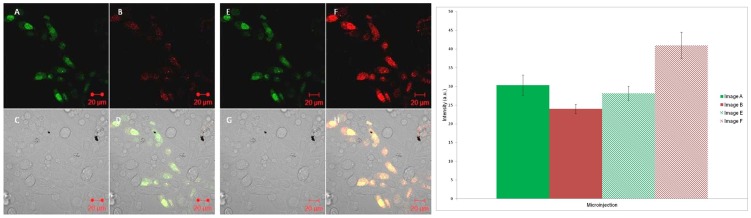
Microinjection confocal microscopy images. Left: Cy3 and Cy5 tagged DNA duplex (**S1:S2**) added to cells via microinjection and imaged using confocal microscopy. Images A/E represents the Cy3 channel; B/F the Cy5 channel; C/G the bright field channel and D/H an overlay of all the channels. Images A–D are excited with the 543 nm laser. Images E–H are excited with both the 543 and 633 nm lasers. Right: Intracellular fluorescence intensity from images A/B and E/F. Data are expressed as mean ± s.e.m from at least ten cells (p<0.001).

Cy3 and Cy5 oligonucleotides **S1** and **S2** were then added to cells *via* microinjection as a preformed duplex. Under these conditions and in contrast to the chemical transfection study, this time when only the Cy3 chromophore was excited using a 543 nm laser, a signal was observed in the Cy5 channel, confirming the occurrence of FRET (Image B, Figure 5). Quantitative data in Figure 5 clearly shows significant Cy5 signal compared to Cy3 signal upon excitation at the Cy3 absorption wavelength only (Figure 5, first two bars on chart). Once again the control study involving the microinjection of the Cy5 strand **S2** only and excitation at 543 nm gave a negligible signal, which confirmed that the FRET signal was genuine (Figure S18 in File S1). Other control studies, which included the microinjection of the doubly-tagged **S3** strand and that of a non-complementary strand pair, gave the expected results, with FRET only occurring for the **S3** system (Figures S19-S21 in File S1).

#### 3. Electroporation

**Figure 6:**
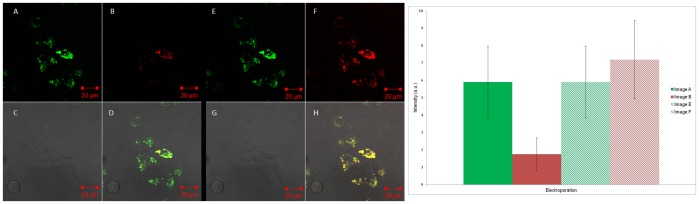
Electroporation confocal microscopy images. Left: Cy3 and Cy5 tagged DNA (**S1:S2**) duplex added to cells via electroporation and imaged using confocal microscopy. Images A/E represents the Cy3 channel; B/F the Cy5 channel the nuclear stain channel; C/G the bright field channel and D/H an overlay of all the channels. Images A–D are excited with a 543 nm laser only. Images E–H are excited with both the 543 and 633 nm lasers. Right: Intracellular fluorescence intensity from images A/B and E/F. Data are expressed as mean ± s.e.m from at least ten cells (p = 0.001 to 0.01).

Cy3 and Cy5 oligonucleotides (**S1** and **S2**) were next added to cells *via* electroporation. The results were similar to the microinjection studies in that when the Cy3 tag in the **S1:S2** duplex was excited using a 543 nm laser, a signal present in the Cy5 channel was observed (Image B, Figure 6) to indicate FRET, which was again supported by control studies including **S2** alone (Figures S22-S23 in File S1). Quantitative analysis of the cell images in Figure 6 confirmed the FRET signal, although the ratio of the Cy5 signal to Cy3 signal was smaller than for microinjection (Figure 6, first two bars on chart). Sequential studies involving the addition of **S1** and **S2** were less conclusive, possibly due at least in part to the damaging effect of physically perturbing the live cell environment more than once. Once again, the controls of adding **S3** and non-complementary strands gave the expected results, with FRET signal observed for the **S3** system only (Figures S24-S25 in File S1). Compared to microinjection, the fluorescence was seen to be not as evenly distributed throughout each cell. This would suggest that the DNA strands show a tendency to accumulate in distinct areas.

## Discussion

The results from the cuvette studies clearly indicate that energy transfer *via* FRET can occur both intramolecularly in the case of **S3** and intermolecularly upon formation of the **S1:S2** duplex. Melting studies confirmed the stability of the duplexes under cell conditions. Furthermore cell lysate studies demonstrate that these systems can in principle remain intact over a period of a few hours if they are not exposed to degrading nucleases. However our results on these systems in cells clearly indicate that the type of technique employed and the status of the cell (fixed or live) have a strong bearing on the degree to which FRET imaging can be successfully observed.

Cells are commonly fixed and permeabilised with alcohols or formaldehyde. However this is incompatible with live cell imaging and the effect of fixation on DNA in cells is uncertain. Nevertheless, these studies clearly indicate that DNA can easily enter fixed/permeabilised cells, as evidenced by the observation of a strong FRET signal when tagged DNA is added either as a duplex or sequentially. That DNA duplexes of this length can remain intact from either simultaneous or sequential addition to fixed cells is clearly shown from these studies, with no FRET observed when using non-complementary strands under the same conditions.

The transfection of live cells with DNA was certainly found to be more challenging, with generally less material entering compared to fixed cells. Despite these strands being relatively small in size, the hydrophilicity and negative charge of the DNA backbone prevents it from crossing biological membranes of live cells unaided. Although chemical transfection has been reported as being relatively inefficient (<80%) [33], [35] and slow (delivery times ∼ 4 hours), it is well established that lipid-based chemical transfection reagents help to mask the negative charge, which allows binding to the cell membrane, uptake by receptor-mediated endocytosis and deposition into endosomes [36]. Our studies indicate that this technique does indeed facilitate cell transfection of singly or double stranded DNA. However in each case, no FRET signal was observed, even though the respective fluorophores were shown to be co-localised. Furthermore the bright spots of fluorescence from both fluorophores suggest that the DNA is not released from the endocytotic vesicles that are formed, which is consistent with nuclease degradation and supports similar findings in previous studies [12], [33], [37]. This interpretation was supported by repeating the transfection experiments on the **S1**:**S2** duplex and **S3** in the presence of bafilomycin, which is known to block degradation by preventing the acidification of the endosomal vesicles [38], [39]. It was interesting to note that under these conditions, DNA was found to be still internalised into vesicles but no longer degraded, with a FRET signal now observed (Images B/D, Figure S26 in File S1).

In contrast to chemical transfection, degradation of DNA in cells does not appear to be a major issue when microinjection or electroporation is used as the transfection technique. In each case, when the DNA was added, the **S1:S2** duplex and the **S3** single strand were less degraded, as evidenced by the observation of a FRET signal. In the case of microinjection, the fluorescence signal was generally evenly distributed throughout the cell. Microinjection can precisely add a controlled dose of material to a single cell, either to the nucleus or cytoplasm [40], [41]. However as found here, despite the high transfection efficiency, microinjection typically only treats a small proportion of cultured cells and also can lead to physical stress [42]. By comparing the quantitative data in Figures 5 and 6, it can be seen that the FRET efficiency, defined here as the ratio between the Cy5 intensity and Cy3 intensity upon excitation at the Cy3 absorption wavelength only, is approximately halved for electroporation compared to microinjection. Despite this, electroporation is a less cumbersome technique, although under the conditions used here, the cell fluorescence distribution was less uniform than in the case of microinjection, which indicates a possible accumulation of the DNA in vesicles. However, although FRET was not widely observed across a large number of cells, it appears that any vesicles that may form are less primed to degrade the DNA than those formed via the endocytotic pathway.

In conclusion, this work represents a relatively rare example of a controlled study that compares a range of different DNA transfection techniques using both fixed and live cells. The work underlines the issues that surround the stability and viability of DNA delivered into live cells by lipid-based transfection, whether the DNA is single or double stranded. In the field of nucleic acid chemistry, it appears that this technique is a more viable option when using other types of nucleic acid (e.g. siRNA) that are capable of entering the cell intact via endocytotic pathways [43]. Otherwise suitable inhibitors have to be used (e.g. bafilomycin) or chemical modifications to the nucleic acid structure have to be made to mitigate nuclease degradation [44]–[46]. On the other hand, our studies indicate that the techniques of microinjection and electroporation are both viable as alternative methods for transfecting cells with single-stranded or duplex DNA. This work provides a further example of the power of FRET in probing the fate of DNA duplexes in cells and as such is relevant to related hybridisation studies in living cells [47]–[49]. Continued work in this area using different nucleic acids, targets, fluorophores, delivery techniques and conditions will only increase our understanding of how DNA and its derivatives may be delivered into cells efficiently and effectively.

## Supporting Information

File S1 **Figure S1: Oligo S1 HPLC analytical. Figure S2: Oligo S2 HPLC analytical. Figure S3: Oligo S3 HPLC analytical. Figure S4: Oligo S4 HPLC analytical. Figure S5: Oligo S5 HPLC analytical. Figure S6: Duplex S1:S2 in cell lysate study.** No change in fluorescence is observed after S1:S2 is incubated in cell lysate at 37°C for two hours. The FRET peak at approximately 660 nm is reduced significantly after the duplex S1:S2 has been incubated with DNase for two hours. Excitation wavelength 554 nm. **Figure S7: Doubly labelled single strand S3 in cell lysate study.** No change in fluorescence is observed after S3 is incubated in cell lysate at 37°C for two hours. The FRET peak at approximately 660 nm disappears after S3 has been incubated with DNase for two hours. Excitation wavelength 554 nm. **Figure S8: Images of single stranded Cy5 tagged DNA (S2) and single stranded Cy3 tagged DNA (S1) added to fixed/permeabilised cells respectively. Figure S9: Images of complementary Cy3 and Cy5 tagged DNA (S1 and S2) added sequentially to fixed/permeabilised cells. Figure S10: Images of Cy3 and Cy5 tagged probe DNA (S3) added to fixed/permeabilised cells. Figure S11: Images of non-complementary Cy3 and Cy5 tagged DNA (S4:S5) added together and non-complementary Cy3 and Cy5 tagged DNA (S4 and S5) added sequentially to fixed/permeabilised cells respectively. Figure S12: Mean emission spectra of regions of interest in methanol fixed cells treated with S1:S2 duplex and S3.** Cells were excited with 543 nm laser only. Therefore, the peak at ca. 670 nm indicates FRET between the Cy3 and Cy5 fluorophores, hence S1:S2 and S3 are intact. Imaging was carried out using spectral imaging inverted confocal microscopy. Background regions had negligible signal. Minimum of ten cells analysed. **Figure S13. Emission spectra of tagged DNA after complex formation with lipid based transfection reagent.** Both S1:S2 and S3 are shown to FRET in the presence of Lipofectamine. Conditions as for transfection: 100 µM DNA, Opti-MEM medium (Life Technologies) and Lipofectamine RNAiMAX (Life Technologies). Excitation wavelength 554 nm. **Figure S14: Images of Cy3 and Cy5 tagged probe DNA (S3) added to cells via lipid based transfection. Figure S15: Mean emission spectra of regions of interest in lipid based transfected cells treated with S1:S2 duplex and S3.** Cells were excited with 543 nm laser only. There is no peak at ca. 670 nm which indicates a lack of FRET between the Cy3 and Cy5 fluorophores, hence S1:S2 and S3 are degraded. Imaging was carried out using spectral imaging inverted confocal microscopy. Background regions had negligible signal. Minimum of ten cells analysed. **Figure S16: Images of single stranded Cy5 tagged DNA (S2) and single stranded Cy3 tagged DNA (S1) added to cells via lipid based transfection respectively. Figure S17: Images of non-complementary Cy3 and Cy5 tagged DNA (S4:S5) added together to cells via lipid based transfection. Figure S18: Images of single stranded Cy5 tagged DNA (S2) added to cells via microinjection. Figure S19: Images of single stranded Cy3 tagged DNA (S1) added to cells via microinjection. Figure S20: Images of Cy3 and Cy5 tagged probe DNA (S3) added to cells via microinjection. Figure S21: Images of non-complementary Cy3 and Cy5 tagged DNA (S4:S5) added together to cells via microinjection. Figure S22: Images of single stranded Cy3 DNA (S1) added to cells via electroporation. Figure S23: Images of single stranded Cy5 DNA (S2) added to cells via electroporation. Figure S24: Images of Cy3 and Cy5 tagged probe DNA (S3) added to cells via electroporation. Figure S25: Images of non-complementary Cy3 and Cy5 tagged DNA (S4:S5) added together to cells via electroporation. Figure S26: Cells treated with bafilomycin upon lipid based transfection of S1:S2 duplex and S3, and imaged using confocal microscopy.** Images A–D are excited with the 543 nm laser only. Images E–H are excited with both the 543 and 633 nm lasers. The top row cells have been treated with the S1:S2 duplex and the bottom row cells have been treated with S3. Images of the Cy5 channel in B and D clearly show a FRET signal. **Table S1: Non-complementary oligonucleotides. Table S2: HPLC retention times. Table S3: Mass spectrometry predicted and actual values. Table S4: Duplex melting temperatures.**
(PDF)
