# Supplementary material for: Electroporation and Microinjection Successfully Deliver Single-Stranded and Duplex DNA into Live Cells as Detected by FRET Measurements
Source: PLoS One. 2014 Apr 22;9(4):e95097. doi: 10.1371/journal.pone.0095097 (PMC3995676; doi:10.1371/journal.pone.0095097)
Supplement: File S1 — Figure S1: Oligo S1 HPLC analytical. Figure S2: Oligo S2 HPLC analytical. Figure S3: Oligo S3 HPLC analytical. Figure S4: Oligo S4 HPLC analytical. Figure S5: Oligo S5 HPLC analytical. Figure S6: Duplex S1:S2 in cell lysate study. No change in fluorescence is observed after S1:S2 is incubated in cell lysate at 37°C for two hours. The FRET peak at approximately 660 nm is reduced significantly after the duplex S1:S2 has been incubated with DNase for two hours. Excitation wavelength 554 nm. Figure S7: Doubly labelled single strand S3 in cell lysate study. No change in fluorescence is observed after S3 is incubated in cell lysate at 37°C for two hours. The FRET peak at approximately 660 nm disappears after S3 has been incubated with DNase for two hours. Excitation wavelength 554 nm. Figure S8: Images of single stranded Cy5 tagged DNA (S2) and single stranded Cy3 tagged DNA (S1) added to fixed/permeabilised cells respectively. Figure S9: Images of complementary Cy3 and Cy5 tagged DNA (S1 and S2) added sequentially to fixed/permeabilised cells. Figure S10: Images of Cy3 and Cy5 tagged probe DNA (S3) added to fixed/permeabilised cells. Figure S11: Images of non-complementary Cy3 and Cy5 tagged DNA (S4:S5) added together and non-complementary Cy3 and Cy5 tagged DNA (S4 and S5) added sequentially to fixed/permeabilised cells respectively. Figure S12: Mean emission spectra of regions of interest in methanol fixed cells treated with S1:S2 duplex and S3. Cells were excited with 543 nm laser only. Therefore, the peak at ca. 670 nm indicates FRET between the Cy3 and Cy5 fluorophores, hence S1:S2 and S3 are intact. Imaging was carried out using spectral imaging inverted confocal microscopy. Background regions had negligible signal. Minimum of ten cells analysed. Figure S13. Emission spectra of tagged DNA after complex formation with lipid based transfection reagent. Both S1:S2 and S3 are shown to FRET in the presence of Lipofectamine. Conditions as for transfection: 100 µM DNA, Op [file pone.0095097.s001.pdf]

Supplementary Information

General Experimental Information:

Unless otherwise stated, solvents and reagents were obtained from commercial suppliers and used without further purification.

Automated DNA synthesis was performed on an Applied Biosystems ABI 394 Synthesiser.

HPLC-grade and DNase-free H_2O was used in all syntheses and studies of oligonucleotides.

Mass spectra of the oligonucleotide sequences were determined on a Waters LCT ESI-TOF mass spectrometer.

HPLC preparative purification was carried out using a Dionex UVD1705 with Phenomenex Clarity 5u Oligo-RP column 150x10 mm. HPLC analytical purification was carried out using a Shimadzu UFLC with Phenomenex Clarity 5u Oligo-RP column 150x4.6 mm.

DNA melting temperatures were determined on a Varian Cary 5000 with a peltier heating accessory on a range of 15 to 85 °C with a heating range of 0.5 °C/min. The value of T_m was calculated from the first derivative of the melting curve using Varian software. Unless stated otherwise, all samples were monitored at 260 nm.

UV/Vis spectra were recorded using a UV-1800 Shimadzu UV spectrophotometer. Fluorescence titrations were carried out on a Shimadzu RF-5301 PC spectrofluorimeter.

Cell lysate was extracted using 2% NP-40, 20 mM Tris pH 7.5 (Fischer), 300 mM NaCl, 2 mM EDTA, 0.02% sodium azide, 1% protease inhibitors (Roche). Chemicals from Sigma Aldrich unless stated.

Random Oligonucleotides

Two further oligonucleotides were synthesised which are not complementary and were used as controls (to show specificity). They are fully characterised below.

Table S1: Non-complementary oligonucleotides

Oligonucleotide Name	Sequence (5' to 3')
**S4**	Cy3-TTTTTTTTTTTTTTTT
**S5**	Cy5- CATTGAGTGAGTCCA

HPLC analytical

Conditions (Thiol Ferro method):

Solvent system C: MeCN; Solvent system D: 0.1 M TEAA pH 7.0

Gradient (linear increase): 0-25 mins, 5%-25% C; 25-35 mins, 25% C hold; 35-40 mins, 100% C; 40-50 mins, 5% C.

Conditions (Oligo analytical method):

Solvent system A: 5% MeCN, 0.1 M TEAA pH 7.0; Solvent system B: 15% MeCN, 0.1 M TEAA pH 7.0; Solvent system C: MeCN

Gradient (linear increase): 0 - 25 mins, 30% B - 50% B; 35 - 45 mins, 0% - 100% C; 45 - 55 mins, 100% C hold; 55-60 mins, 30% B.

<u>Conditions (DMT-on method):</u>

Solvent system B: 15% MeCN, 0.1 M TEAA pH 7.0; Solvent system C: MeCN

Gradient (linear increase): 0 - 10 mins, 100% B; 20 - 25 mins, 70% B; 25 - 35 mins, 100% C hold; 35 - 36 mins, 100% B; 36 - 41 mins, 100% B hold.

30 µl oligonucleotide samples, *ca.* 70 µM was auto injected. Flow rate, 1.0 ml/min, monitored at 260 nm.

Table S2: HPLC retention times

Oligonucleotide Name	Sequence (5' to 3')	Retention Time (mins)
**S1**	Cy3-TGGACTCTCTCAATG	18.795
**S2**	Cy5-CATTGAGAGAGTCCA	30.614
**S3**	Cy5-TGGACTCTCTCAATG-Cy3	21.323
**S4**	Cy3-TTTTTTTTTTTTTTT	40.384
**S5**	Cy5- CATTGAGTGAGTCCA	30.303

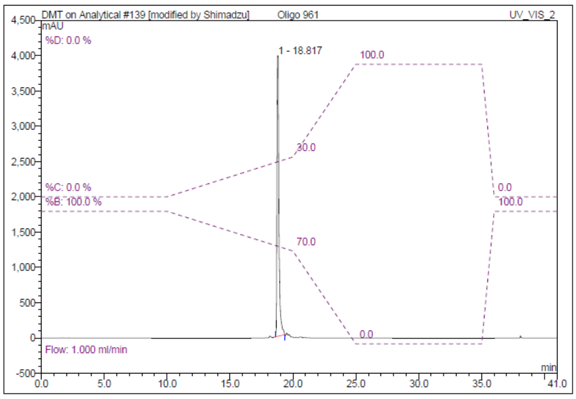

Figure S1: Oligo S1 HPLC analytical

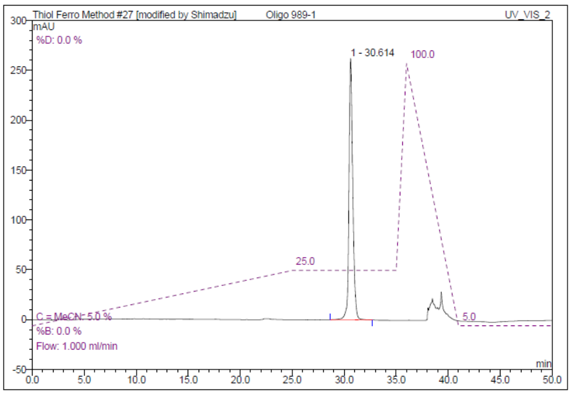

Figure S2: Oligo S2 HPLC analytical

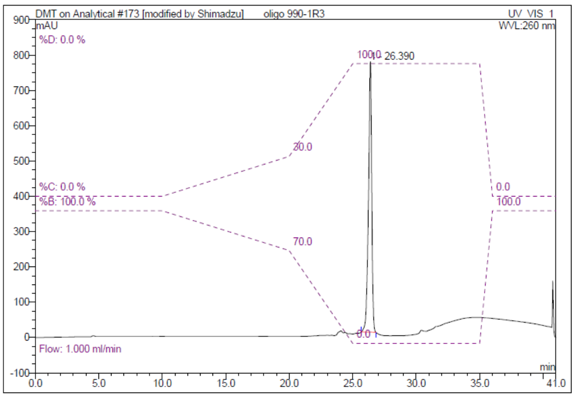

Figure S3: Oligo S3 HPLC analytical

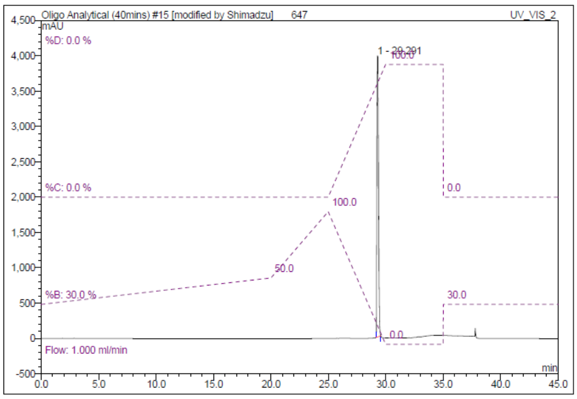

Figure S4: Oligo S4 HPLC analytical

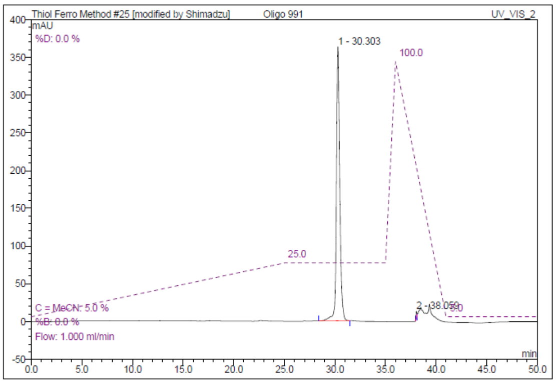

Figure S5: Oligo S5 HPLC analytical

Mass Spectrometry

Table S3: Mass spectrometry predicted and actual values

Oligonucleotide Name	Sequence (5' to 3')	Predicted Mass	Actual Mass
**S1**	Cy3-TGGACTCTCTCAATG	5050	5050
**S2**	Cy5-CATTGAGAGAGTCCA	5134	5133
**S3**	Cy5-TGGACTCTCTCAATG-Cy3	5583	5582
**S4**	Cy3-TTTTTTTTTTTTTTTT	5312	5312
**S5**	Cy5- CATTGAGTGAGTCCA	5125	5124

Melting Temperatures

The melting temperature is defined as the temperature at which 50% of the DNA is part of a duplex and 50% is single-stranded.

Table S4: Duplex melting temperatures (10 mM sodium phosphate, 100 mM NaCl, pH 7.0, 5 µM each DNA strand)

Duplex	Melting Temperature (°C)
**S1:S2**	62
**S1:S2** (unmodified)	56.5

Cell Lysate Cuvette Studies

Cell lysate was extracted using 2% NP-40, 20 mM Tris pH 7.5 (Fischer), 300 mM NaCl, 2 mM EDTA, 0.02% sodium azide, 1% protease inhibitors. Chemicals from Sigma Aldrich unless stated.

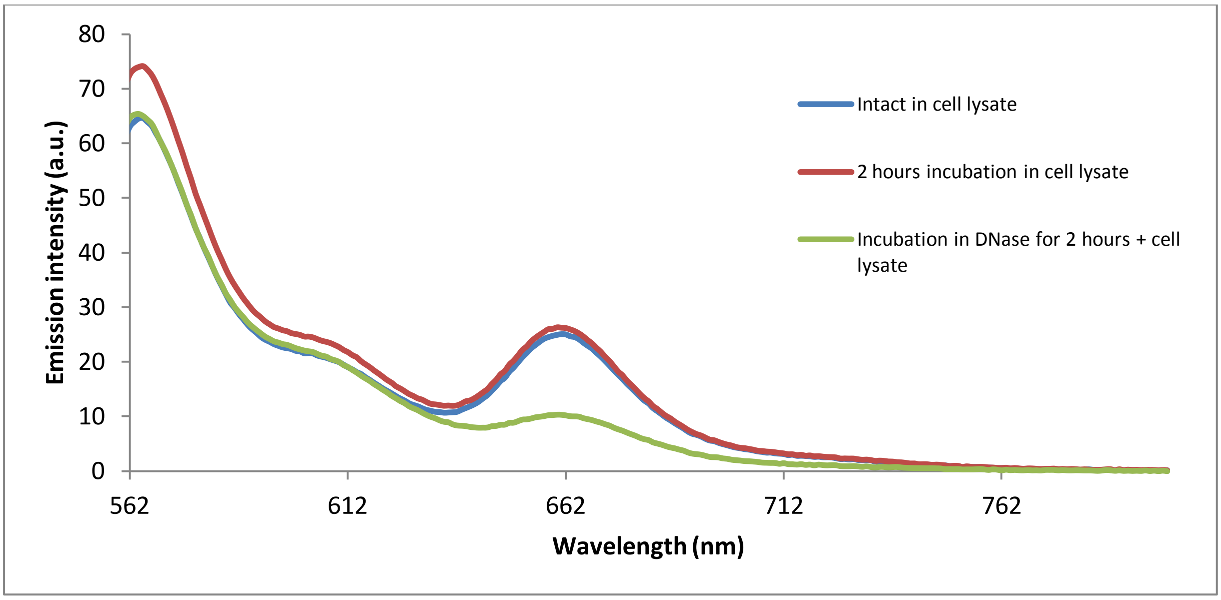

Figure S6: No change in fluorescence is observed after S1:S2 is incubated in cell lysate at 37°C for two hours. The FRET peak at approximately 660 nm is reduced significantly after the duplex S1:S2 has been incubated with DNase for two hours. Excitation wavelength 554 nm.

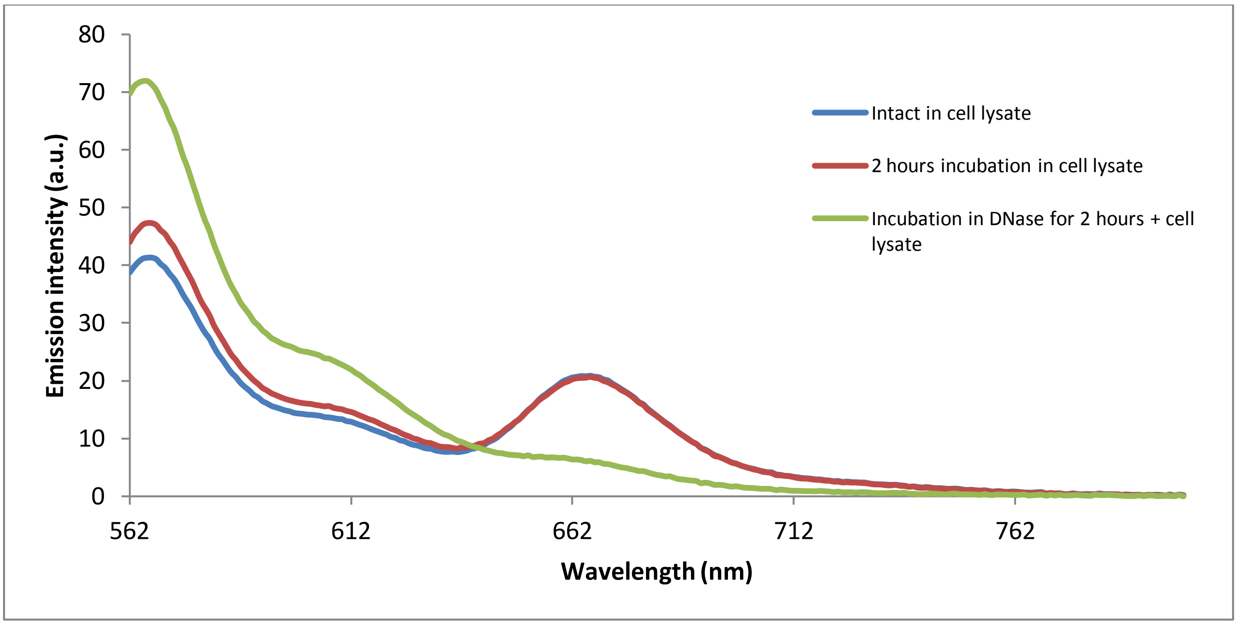

Figure S7: No change in fluorescence is observed after S3 is incubated in cell lysate at 37°C for two hours. The FRET peak at approximately 660 nm disappears after S3 has been incubated with DNase for two hours. Excitation wavelength 554 nm.

Control Cell Experiments

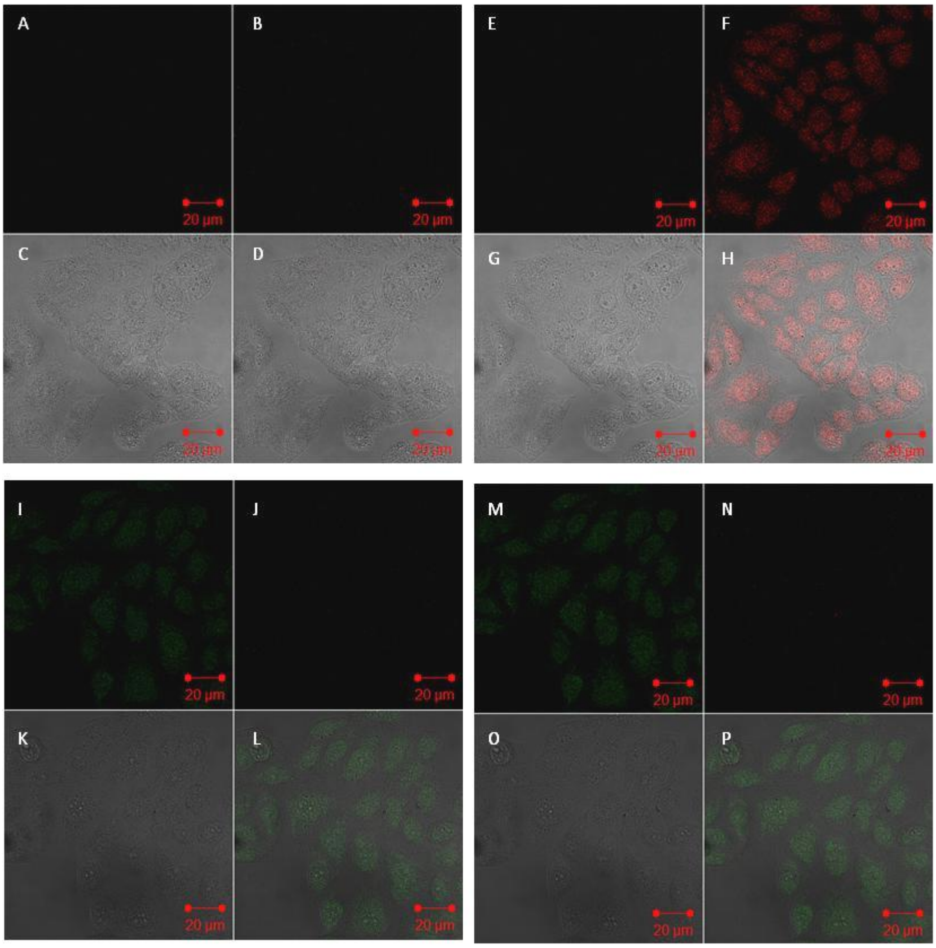

Figure S8: Images A-H show single stranded Cy5 tagged DNA (S2) added to fixed/permeabilised cells and imaged using confocal microscopy. Images A/E represents the Cy3 channel; B/F the Cy5 channel; C/G the bright field channel and D/H an overlay of all the channels. Images A-D are excited with a 543 nm laser only. Images E-H are excited with both the 543 and 633 nm lasers.

Images I-P show single stranded Cy3 tagged DNA (S1) added to fixed/permeabilised cells and imaged using confocal microscopy. Images I/M represents the Cy3 channel; J/N the Cy5 channel; K/O the bright field channel and L/P an overlay of all the channels. Images I-L are excited with a 543 nm laser only. Images M-P are excited with both the 543 and 633 nm lasers.

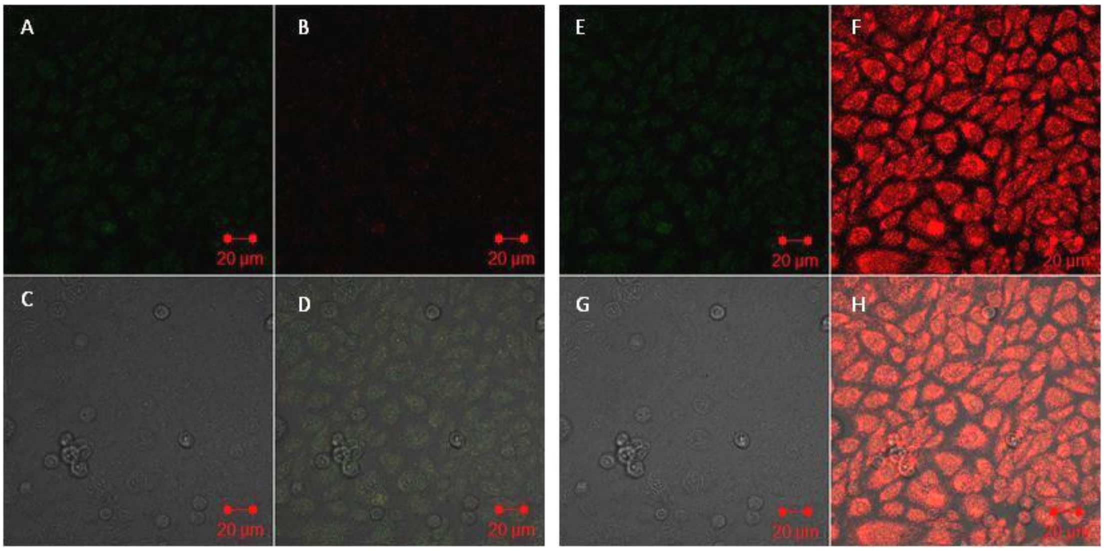

Figure S9: Images A-H show complementary Cy3 and Cy5 tagged DNA (S1 and S2) added sequentially to fixed/permeabilised cells imaged using confocal microscopy. Images A/E represents the Cy3 channel; B/F the Cy5 channel; C/G the bright field channel and D/H an overlay of all the channels. Images A-D are excited with a 543 nm laser only. Images E-H are excited with both the 543 and 633 nm lasers.

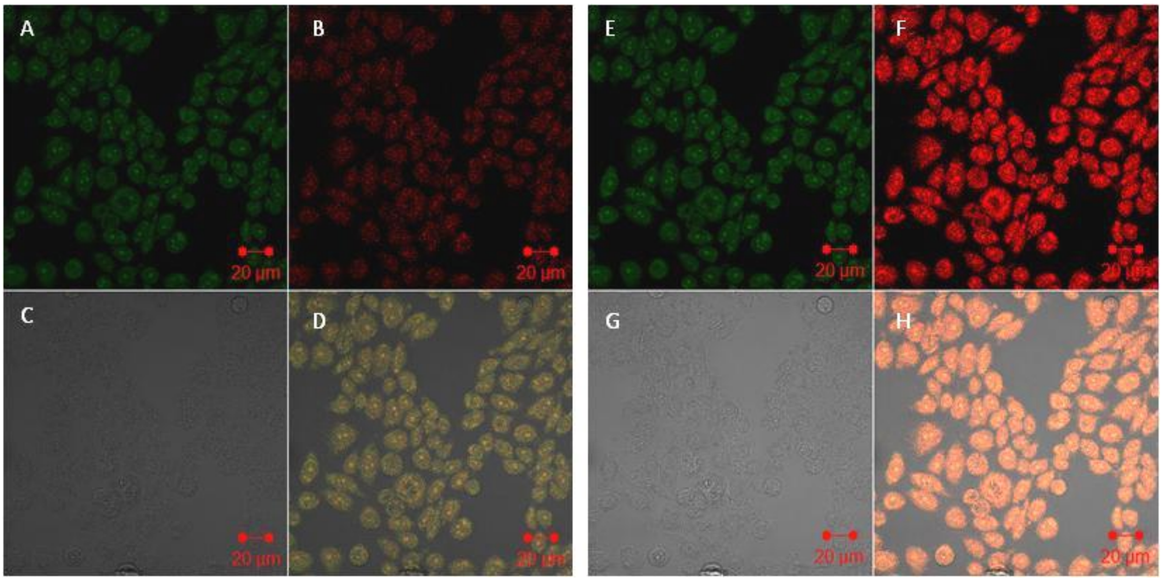

Figure S10: Images A-H show Cy3 and Cy5 tagged probe DNA (S3) added to fixed/permeabilised cells imaged using confocal microscopy. Images A/E represents the Cy3 channel; B/F the Cy5 channel; C/G the bright field channel and D/H an overlay of all the channels. Images A-D are excited with a 543 nm laser only. Images E-H are excited with both the 543 and 633 nm lasers.

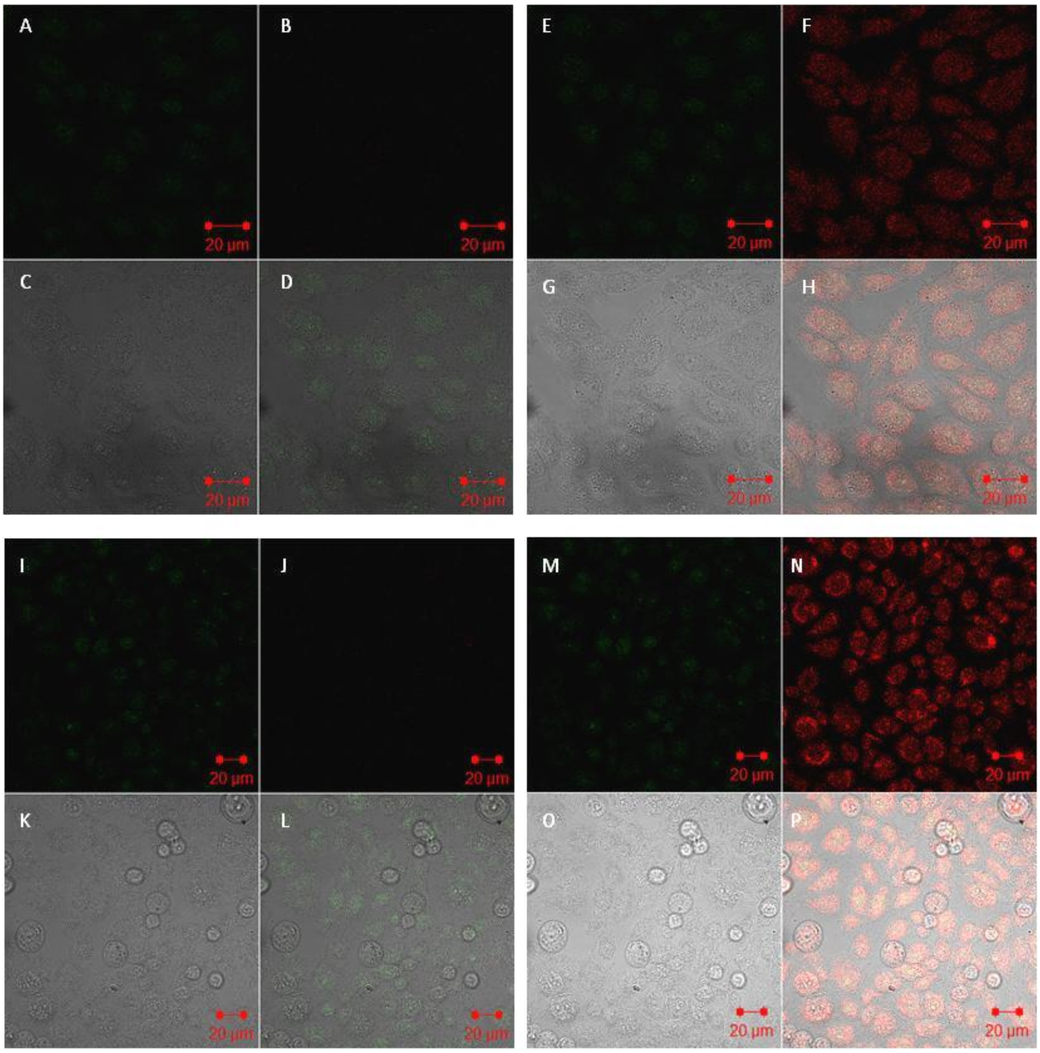

Figure S11: Images A-H show non-complementary Cy3 and Cy5 tagged DNA (S4:S5) added together to fixed/permeabilised cells and imaged using confocal microscopy. Images A/E represents the Cy3 channel; B/F the Cy5 channel; C/G the bright field channel and D/H an overlay of all the channels. Images A-D are excited with a 543 nm laser only. Images E-H are excited with both the 543 and 633 nm lasers.

Images I-P show non-complementary Cy3 and Cy5 tagged DNA (S4 and S5) added sequentially to fixed/permeabilised cells imaged using confocal microscopy. Images I/M represents the Cy3 channel; J/N the Cy5 channel; K/O the bright field channel and L/P an overlay of all the channels. Images I-L are excited with a 543 nm laser only. Images M-P are excited with both the 543 and 633 nm lasers.

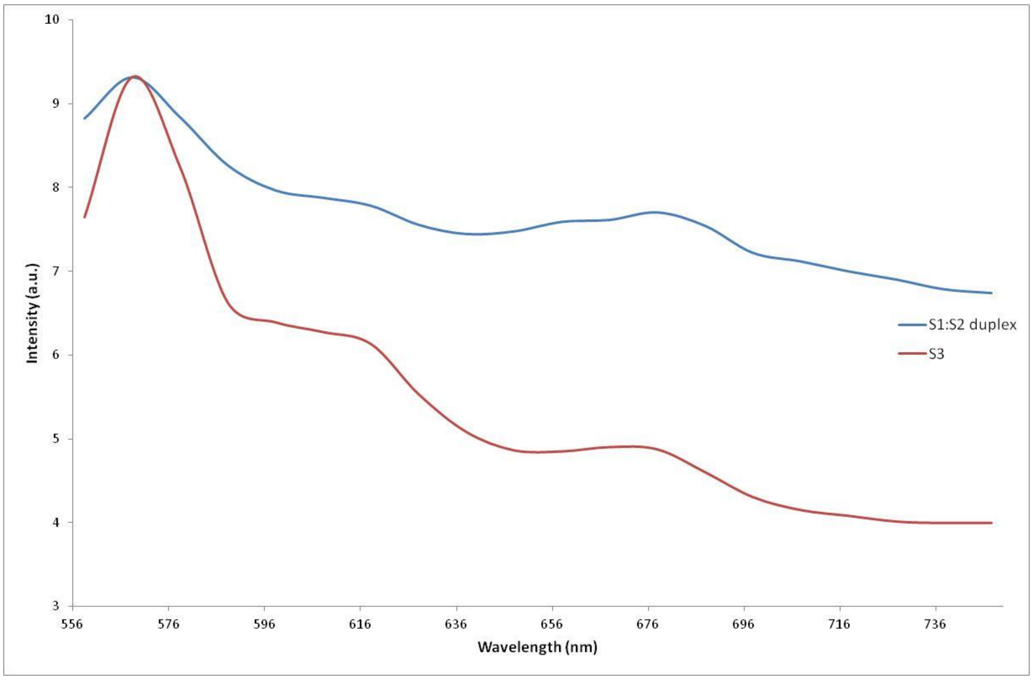

Figure S12: Mean emission spectra of regions of interest in methanol fixed cells treated with S1:S2 duplex and S3. Cells were excited with 543 nm laser only. Therefore, the peak at ca. 670 nm indicates FRET between the Cy3 and Cy5 fluorophores, hence S1:S2 and S3 are intact. Imaging was carried out using spectral imaging inverted confocal microscopy. Background regions had negligible signal. Minimum of ten cells analysed.

Complex Formation with Lipofectamine

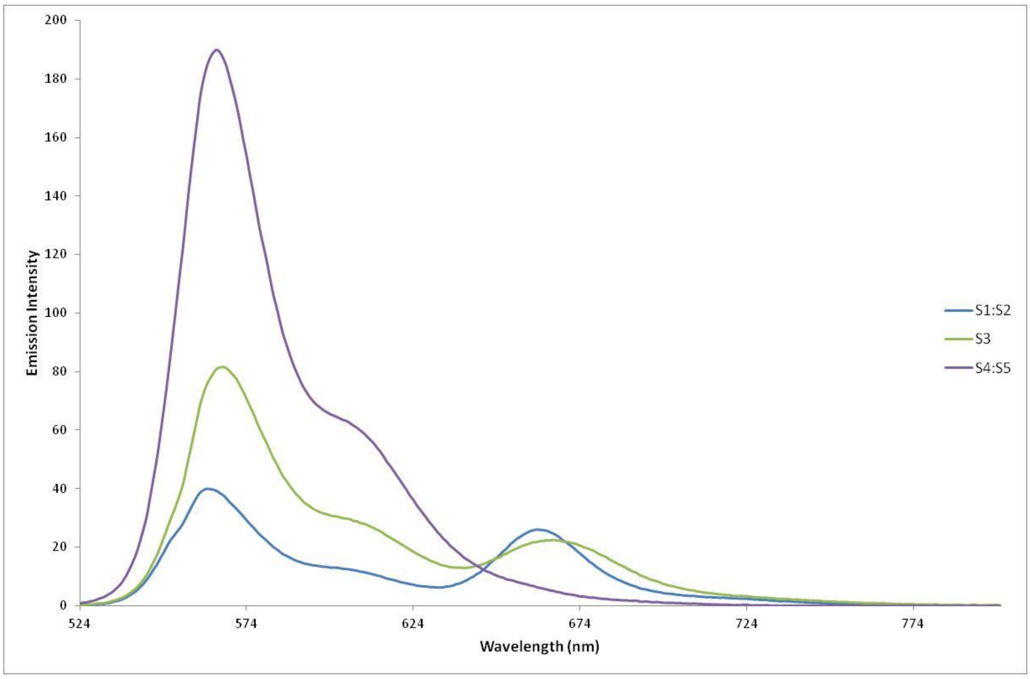

Figure S13: Emission spectra of tagged DNA after complex formation with lipid based transfection reagent. Both S1:S2 and S3 are shown to FRET in the presence of Lipofectamine. Conditions as for transfection: 100 µM DNA, Opti-MEM medium (Life Technologies) and Lipofectamine RNAiMAX (Life Technologies). Excitation wavelength 554 nm.

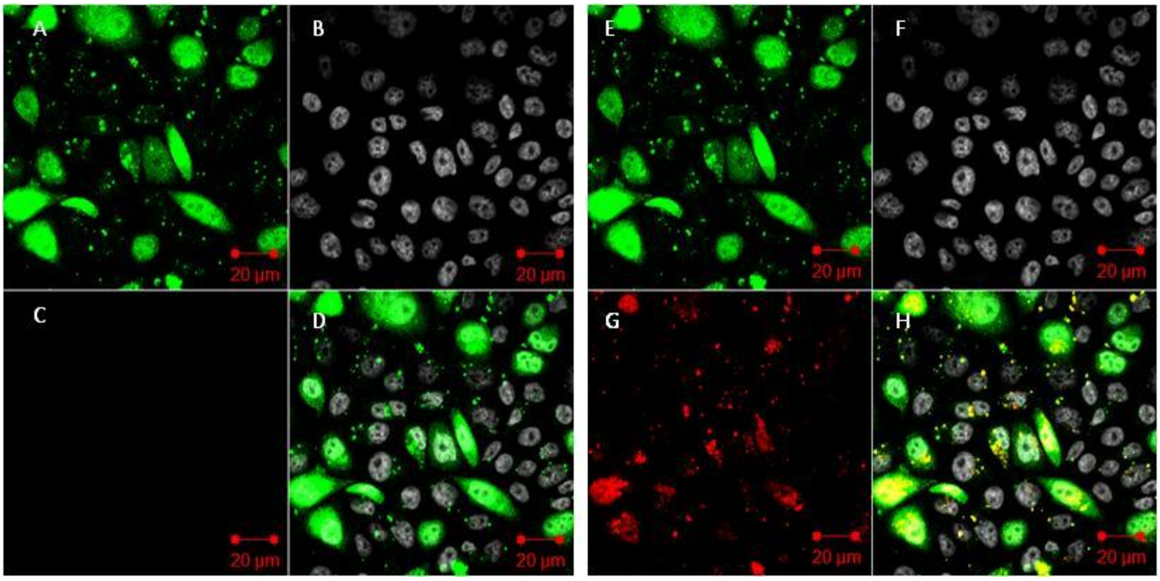

Figure S14: Images A-H show Cy3 and Cy5 tagged probe DNA (S3) added to cells via lipid based transfection and imaged using confocal microscopy. Images A/E represents the Cy3 channel; B/F the nuclear stain channel; C/G the Cy5 channel and D/H an overlay of all the channels. Images A-D are excited with a 543 nm laser only. Images E-H are excited with both the 543 and 633 nm lasers.

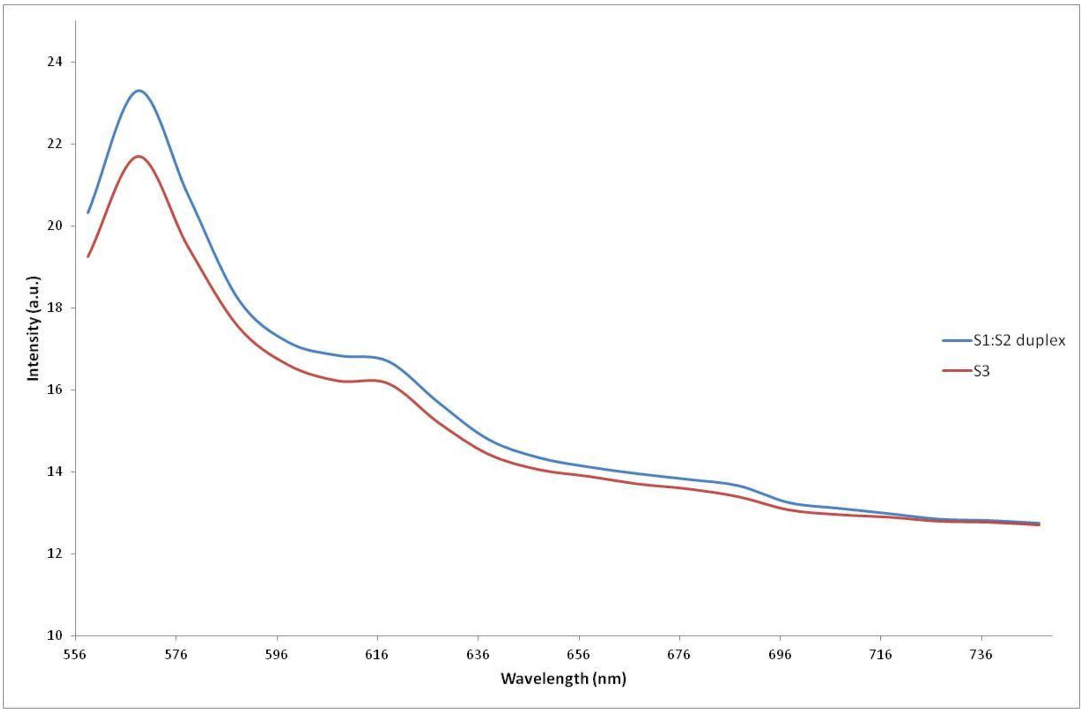

Figure S15: Mean emission spectra of regions of interest in lipid based transfected cells treated with S1:S2 duplex and S3. Cells were excited with 543 nm laser only. There is no peak at ca. 670 nm which indicates a lack of FRET between the Cy3 and Cy5 fluorophores, hence S1:S2 and S3 are degraded. Imaging was carried out using spectral imaging inverted confocal microscopy. Background regions had negligible signal. Minimum of ten cells analysed.

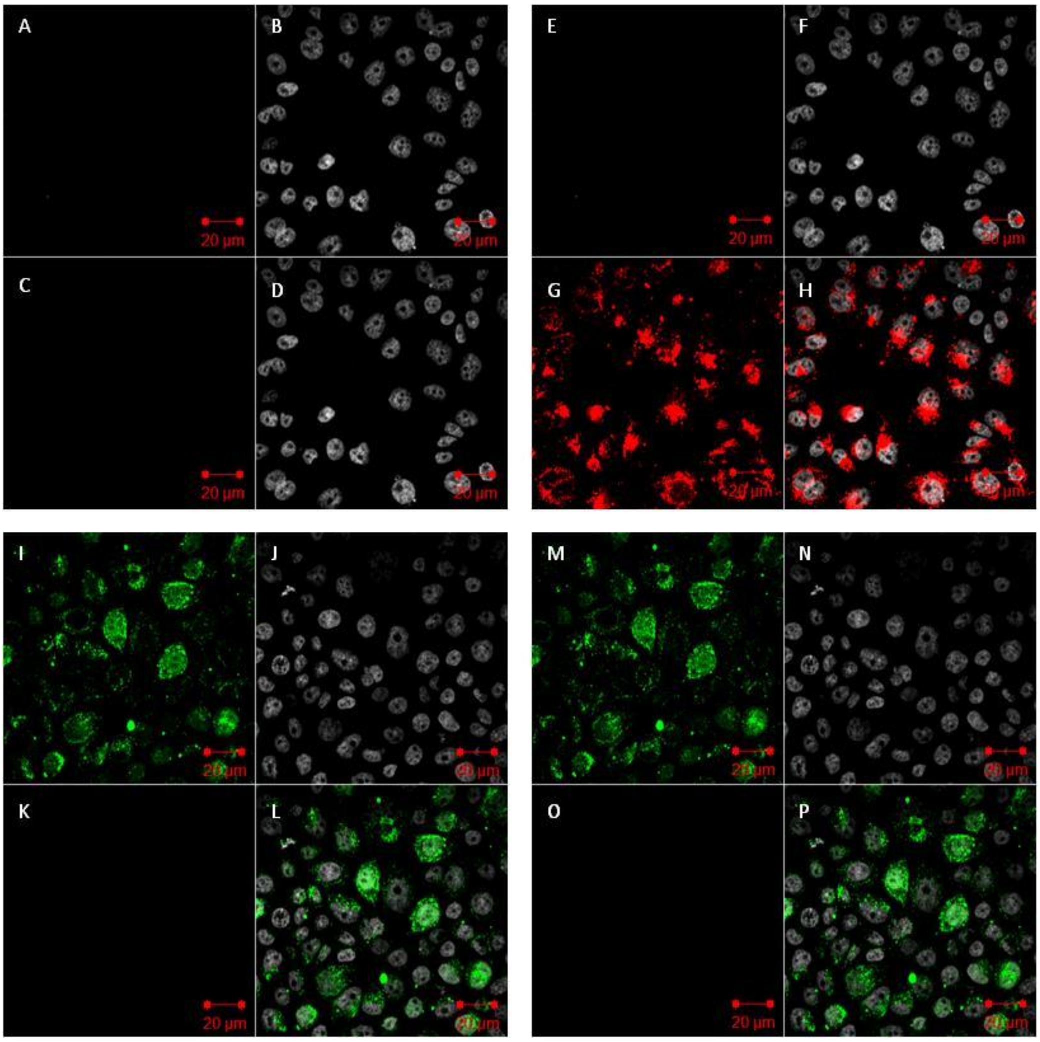

Figure S16: Images A-H show single stranded Cy5 tagged DNA (S2) added to cells via lipid based transfection and imaged using confocal microscopy. Images A/E represents the Cy3 channel; B/F the nuclear stain channel; C/G the Cy5 channel and D/H an overlay of all the channels. Images A-D are excited with a 543 nm laser only. Images E-H are excited with both the 543 and 633 nm lasers.

Images I-P show single stranded Cy3 tagged DNA (S1) added to cells via lipid based transfection and imaged using confocal microscopy. Images I/M represents the Cy3 channel; J/N the nuclear stain channel; K/O the Cy5 channel and L/P an overlay of all the channels. Images I-L are excited with a 543 nm laser only. Images M-P are excited with both the 543 and 633 nm lasers.

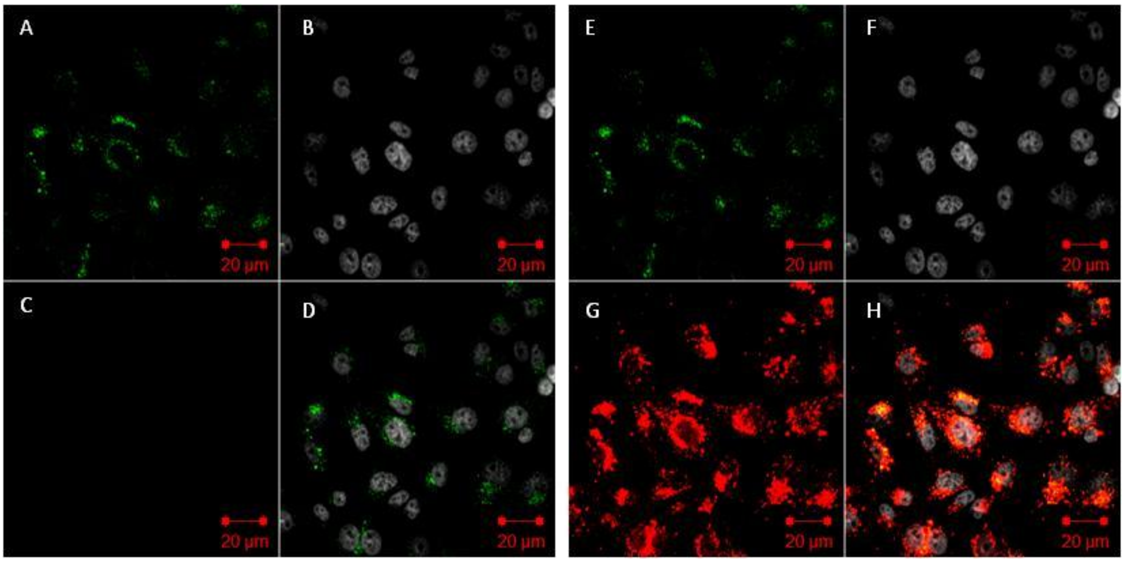

Figure S17: Images A-H show non-complementary Cy3 and Cy5 tagged DNA (S4:S5) added together to cells via lipid based transfection and imaged using confocal microscopy. Images A/E represents the Cy3 channel; B/F the nuclear stain channel; C/G the Cy5 channel and D/H an overlay of all the channels. Images A-D are excited with a 543 nm laser only. Images E-H are excited with both the 543 and 633 nm lasers.

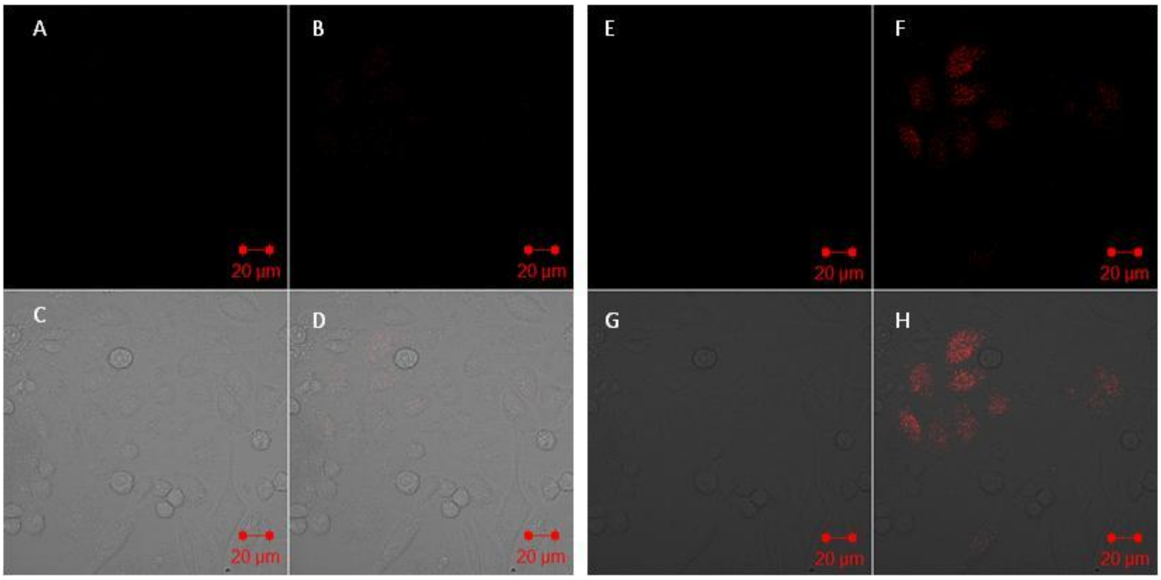

Figure S18: Images A-H show single stranded Cy5 tagged DNA (S2) added to cells via microinjection and imaged using confocal microscopy. Images A/E represents the Cy3 channel; B/F the Cy5 channel; C/G the bright field channel and D/H an overlay of all the channels. Images A-D are excited with a 543 nm laser only. Images E-H are excited with both the 543 and 633 nm lasers.

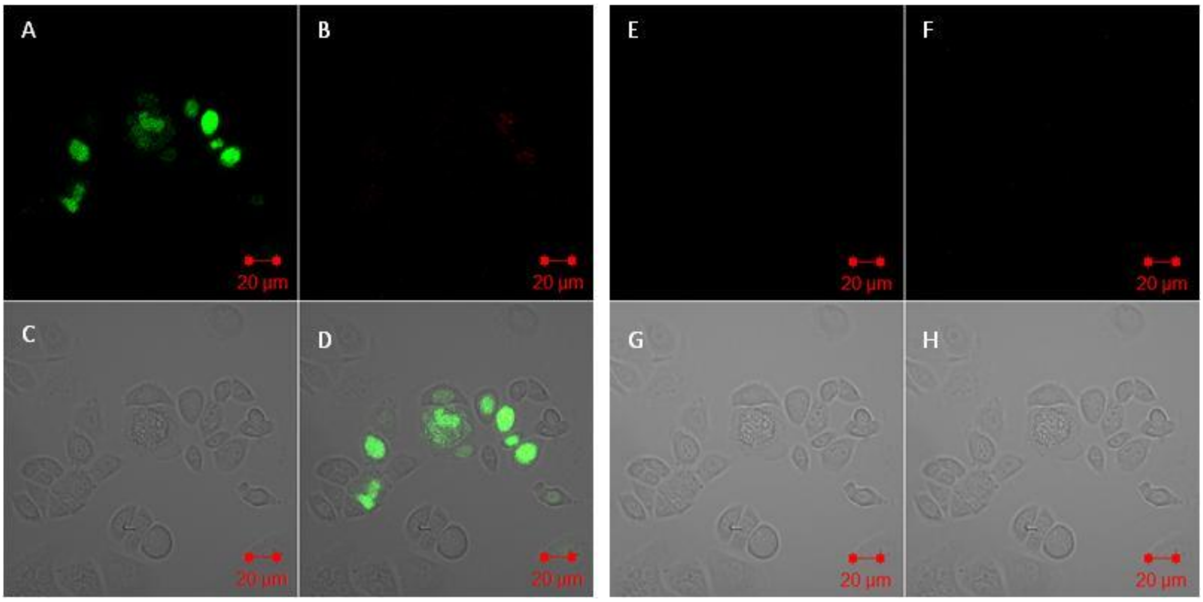

Figure S19: Images A-H show single stranded Cy3 tagged DNA (S1) added to cells via microinjection and imaged using confocal microscopy. Images A/E represents the Cy3 channel; B/F the Cy5 channel; C/G the bright field channel and D/H an overlay of all the channels. Images A-D are excited with a 543 nm laser only. Images E-H are excited with both the 543 and 633 nm lasers.

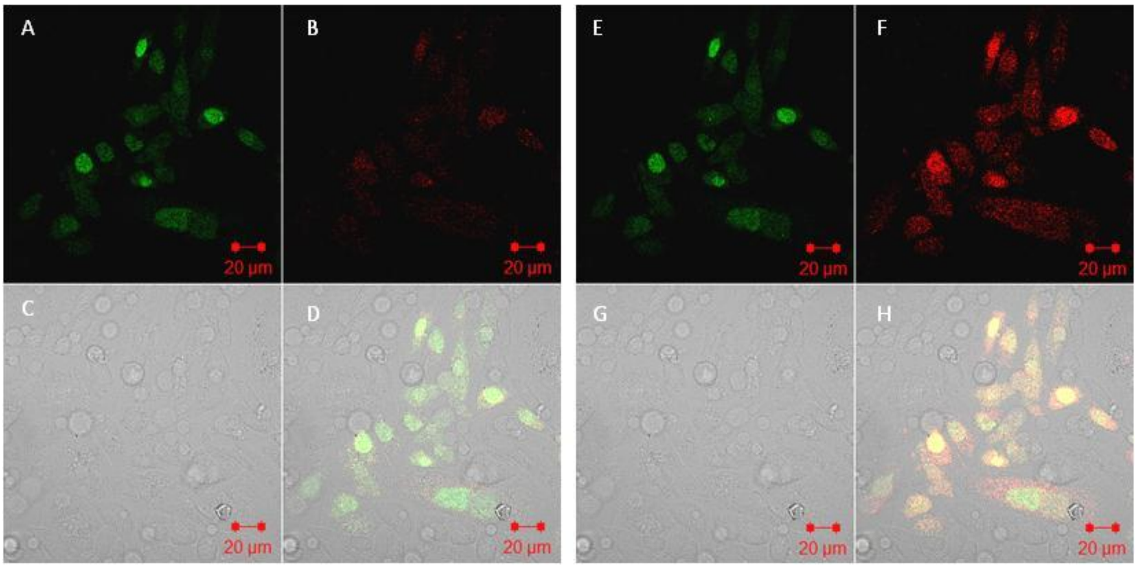

Figure S20: Images A-H show Cy3 and Cy5 tagged probe DNA (S3) added to cells via microinjection and imaged using confocal microscopy. Images A/E represents the Cy3 channel; B/F the Cy5 channel; C/G the bright field channel and D/H an overlay of all the channels. Images A-D are excited with a 543 nm laser only. Images E-H are excited with both the 543 and 633 nm lasers.

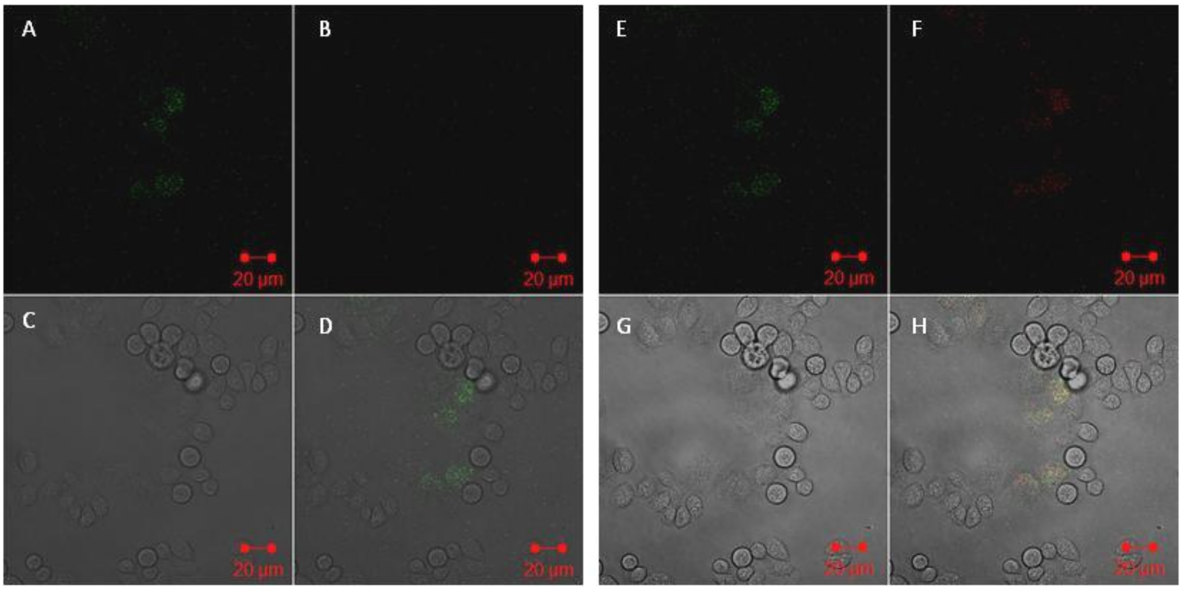

Figure S21: Images A-H show non-complementary Cy3 and Cy5 tagged DNA (S4:S5) added together to cells via microinjection and imaged using confocal microscopy. Images A/E represents the Cy3 channel; B/F the Cy5 channel; C/G the bright field channel and D/H an overlay of all the channels. Images A-D are excited with a 543 nm laser only. Images E-H are excited with both the 543 and 633 nm lasers.

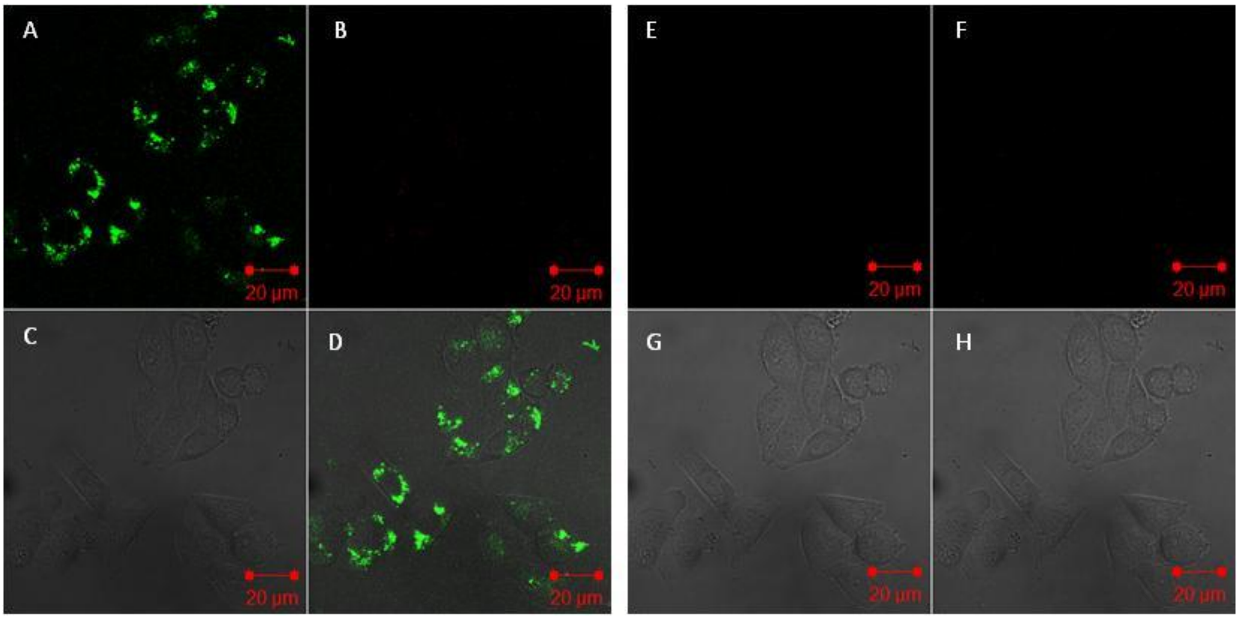

Figure S22: Images A-H show single stranded Cy3 DNA (S1) added to cells via electroporation and imaged using confocal microscopy. Images A/E represents the Cy3 channel; B/F the Cy5 channel; C/G the bright field channel and D/H an overlay of all the channels. Images A-D are excited with a 543 nm laser only. Images E-H are excited with a 633 nm laser only.

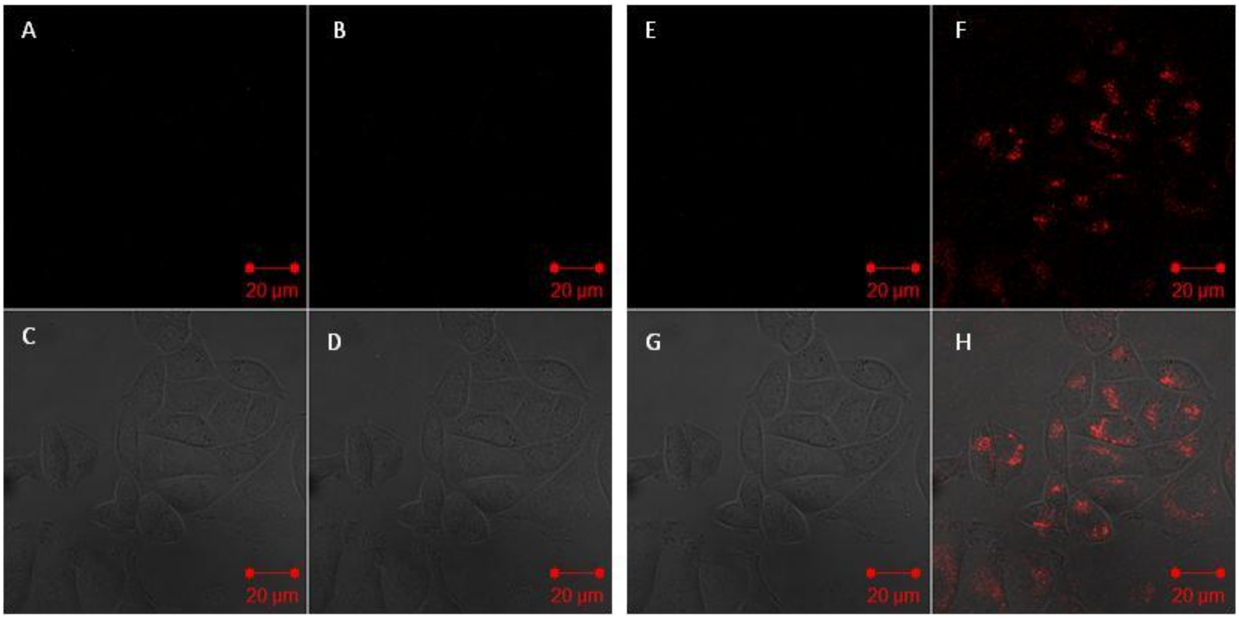

Figure S23: Images A-H show single stranded Cy5 DNA (S2) added to cells via electroporation and imaged using confocal microscopy. Images A/E represents the Cy3 channel; B/F the Cy5 channel; C/G the bright field channel and D/H an overlay of all the channels. Images A-D are excited with a 543 nm laser only. Images E-H are excited with a 633 nm laser only.

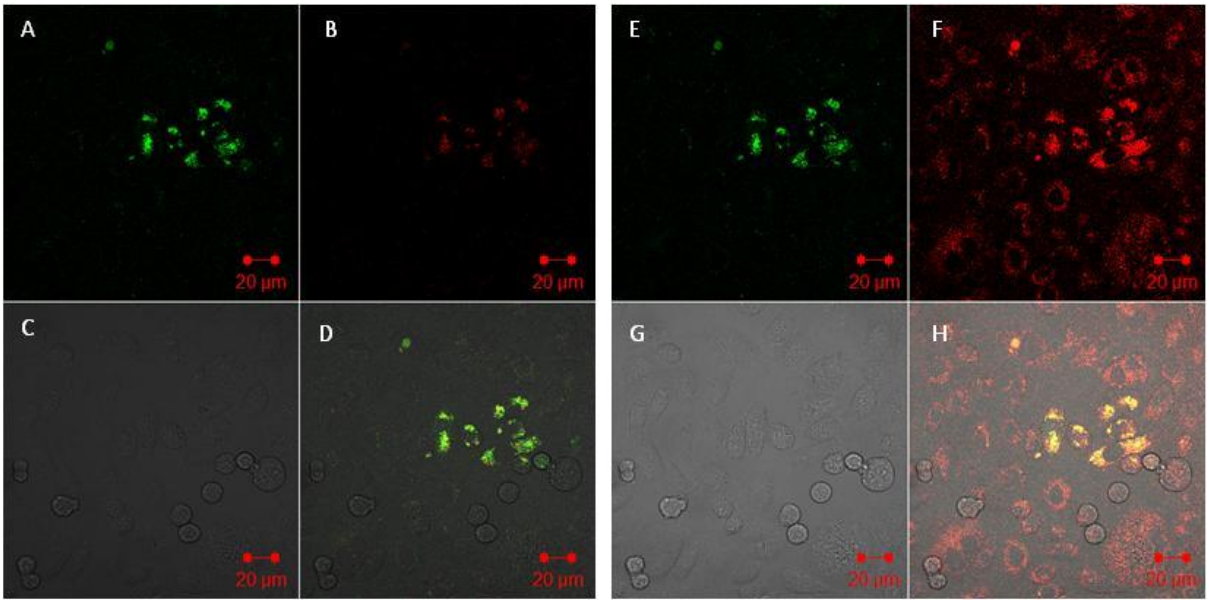

Figure S24: Images A-H show Cy3 and Cy5 tagged probe DNA (S3) added to cells via electroporation and imaged using confocal microscopy. Images A/E represents the Cy3 channel; B/F the Cy5 channel; C/G the bright field channel and D/H an overlay of all the channels. Images A-D are excited with a 543 nm laser only. Images E-H are excited with both the 543 and 633 nm lasers.

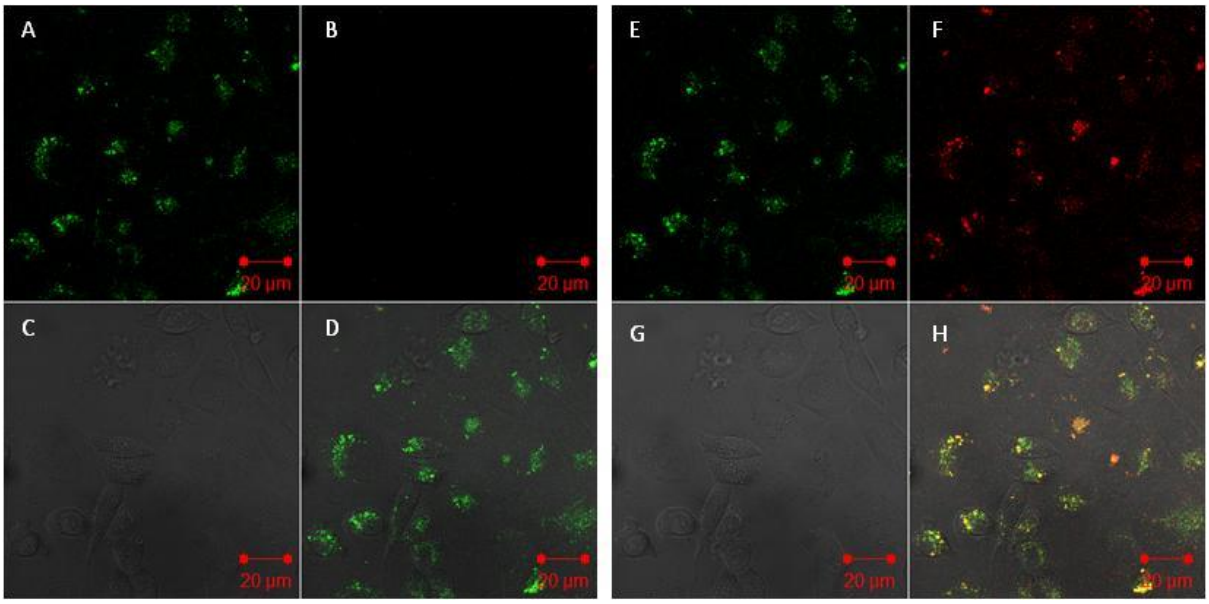

Figure S25: Images A-H show non-complementary Cy3 and Cy5 tagged DNA (S4:S5) added together to cells via electroporation and imaged using confocal microscopy. Images A/E represents the Cy3 channel; B/F the Cy5 channel; C/G the bright field channel and D/H an overlay of all the channels. Images A-D are excited with a 543 nm laser only. Images E-H are excited with both the 543 and 633 nm lasers.

Blocking degradation by Bafilomycin

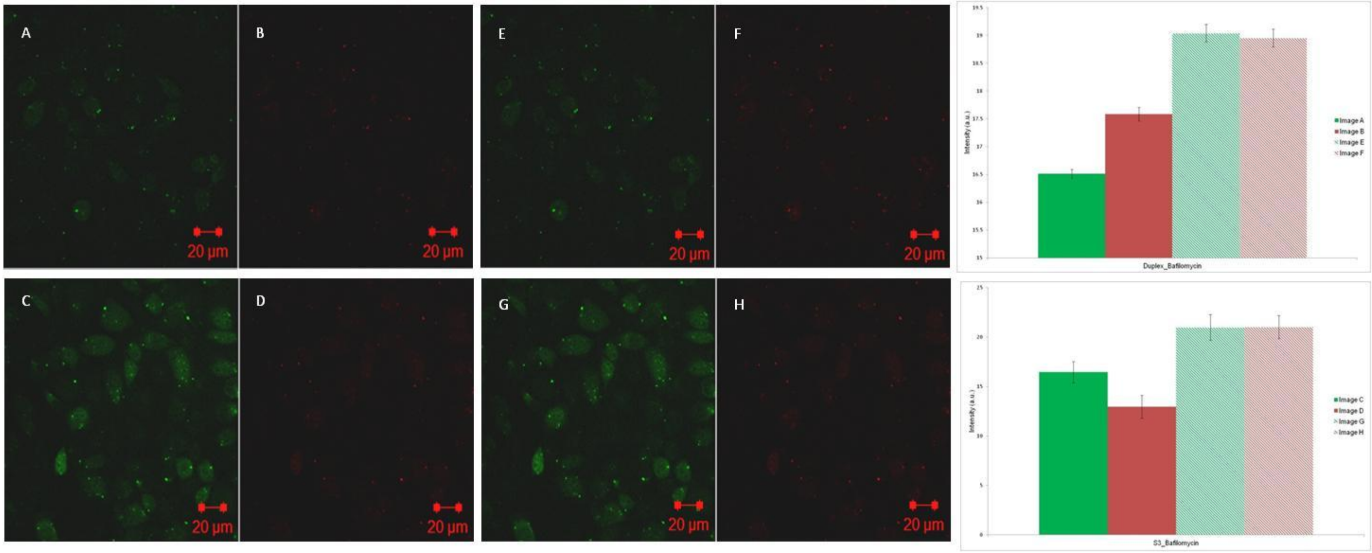

Figure S26: Cells treated with bafilomycin upon lipid based transfection of S1:S2 duplex and S3, and imaged using confocal microscopy. Images A-D are excited with the 543 nm laser only. Images E-H are excited with both the 543 and 633 nm lasers. The top row cells have been treated with the S1:S2 duplex and the bottom row cells have been treated with S3. Images of the Cy5 channel in B and D clearly show a FRET signal.
